# Supplementary material for: Cerebellar Cells Self-Assemble into Functional Organoids on Synthetic, Chemically Crosslinked ECM-Mimicking Peptide Hydrogels
Source: Biomolecules. 2020 May 12;10(5):754. doi: 10.3390/biom10050754 (PMC7277677; doi:10.3390/biom10050754)
Supplement: Supplementary file 1 [file biomolecules-10-00754-s001.pdf]

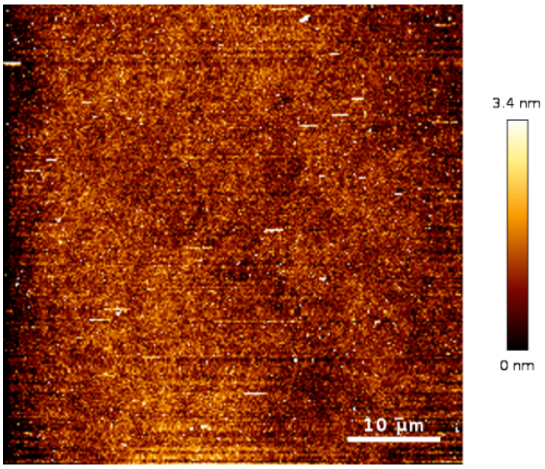

Supplementary Figure S1. Representative AFM topography image of a glass surface treated with (3-aminopropyl) trimethoxy silane (APTMS) and subsequently modified with glutaraldehyde (GA). The image was recorded in PBS pH 7.4 buffer solution.
